# Analysis of Tospovirus NSs Proteins in Suppression of Systemic Silencing

**DOI:** 10.1371/journal.pone.0134517

**Published:** 2015-08-14

**Authors:** Marcio Hedil, Mark G. Sterken, Dryas de Ronde, Dick Lohuis, Richard Kormelink

**Affiliations:** Laboratory of Virology, Department of Plant Sciences, Wageningen University, Wageningen, the Netherlands; Leibniz-Institute for Vegetable and Ornamental Crops, GERMANY

## Abstract

RNA silencing is a sequence-specific gene regulation mechanism that in plants also acts antiviral. In order to counteract antiviral RNA silencing, viruses have evolved RNA silencing suppressors (RSS). In the case of tospoviruses, the non-structural NSs protein has been identified as the RSS. Although the tomato spotted wilt virus (TSWV) tospovirus NSs protein has been shown to exhibit affinity to long and small dsRNA molecules, its ability to suppress the non-cell autonomous part of RNA silencing has only been studied to a limited extent. Here, the NSs proteins of TSWV, groundnut ringspot virus (GRSV) and tomato yellow ring virus (TYRV), representatives for three distinct tospovirus species, have been studied on their ability and strength to suppress local and systemic silencing. A system has been developed to quantify suppression of GFP silencing in *Nicotiana benthamiana* 16C lines, to allow a comparison of relative RNA silencing suppressor strength. It is shown that NSs of all three tospoviruses are suppressors of local and systemic silencing. Unexpectedly, suppression of systemic RNA silencing by NSs^TYRV^ was just as strong as those by NSs^TSWV^ and NSs^GRSV^, even though NSs^TYRV^ was expressed in lower amounts. Using the system established, a set of selected NSs^TSWV^ gene constructs mutated in predicted RNA binding domains, as well as NSs from TSWV isolates 160 and 171 (resistance breakers of the *Tsw* resistance gene), were analyzed for their ability to suppress systemic GFP silencing. The results indicate another mode of RNA silencing suppression by NSs that acts further downstream the biogenesis of siRNAs and their sequestration. The findings are discussed in light of the affinity of NSs for small and long dsRNA, and recent mutant screen of NSs^TSWV^ to map domains required for RSS activity and triggering of *Tsw*-governed resistance.

## Introduction

In plants RNA silencing, besides playing a major role in host gene regulation, is also part of the innate immune system, targeting the nucleic acids of viruses and other molecular parasites, leading to their degradation or translational arrest [1, 2]. In order to counteract the RNA silencing-based defense, plant viruses have evolved RNA silencing suppressor (RSS) proteins [1]. The most common mode of action of viral RSS involves sequestration of the siRNAs [3, 4]. Other viral silencing suppression strategies include targeting dsRNA precursors, therefore preventing their recognition and processing by Dicer (like) proteins [5], or targeting key proteins of the RNA silencing pathway, e.g. the argonaute protein as done by the silencing suppressor 2b protein of cucumber mosaic virus [6, 7].

Systemic silencing is the non-cell autonomous arm of RNA silencing and is also part of the plant innate immune response against viruses [8]. During a viral infection in plants, part of the generated siRNAs become functionally active after being transported via plasmodesmata to neighboring cells or via the phloem in a source-to-sink direction, where they activate the silencing response in naive cells [9, 10]. Although the exact composition of the mobile RNA silencing signal responsible for the movement of RNA silencing is not fully known, there is evidence demonstrating the involvement of 21 and/or 22 nt-sized siRNAs in this signal [11, 12]. Viral proteins that exert RSS activity by sequestering siRNAs, not only prevent their uploading into RISC but also the spread of the systemic silencing signal, and as a result enhance the establishment of systemic infection [13, 14].

Tospoviruses are the plant-pathogenic members of the *Bunyaviridae*, a family that primarily consists of animal-infecting viruses. Like all members of this family, tospoviruses (type species: *Tomato spotted wilt virus*) contain three RNA genome segments of negative/ambisense polarity, denoted Large (L), Medium (M) and Small (S) according to their size. To counteract RNA silencing, TSWV encodes a nonstructural protein (NSs) that exhibits RSS activity [15, 16]. While currently more than 20 distinct tospovirus species have been defined, eight of which confirmed by the ICTV [17, 18], research on tospovirus silencing suppression is primarily limited to TSWV NSs. Silencing-suppressor defective (NSs-mutant) TSWV strains show a clear increase of viral siRNAs (vsiRNAs), specifically of the 21 nt class [19]. Besides being able to suppress local RNA silencing, NSs^TSWV^ has also been briefly reported to suppress systemic silencing [16]. Biochemical analysis has shown that NSs^TSWV^ is able to bind long and small (si- and micro- (mi)) dsRNA, indicating that NSs^TSWV^ likely suppresses RNA silencing by sequestering long and small dsRNAs to respectively prevent cleavage by dicer-like proteins (DCLs) and uploading into RISC [20]. TSWV NSs also contains a WG/GW motif that for several other viral RSS proteins has been reported to facilitate binding to AGO1 and thereby antiviral RISC activity [21]. Although this motif is absent from most other tospovirus NSs proteins, mutation of this motif from TSWV NSs abolishes its local RSS activity [22]. For groundnut bud necrosis virus (GBNV), a distinct Asian tospovirus, the NSs has been reported to contain NTPase and 5’ phosphatase activity, and corresponds with the presence of Walker A and B motifs [23]. Recently, GBNV NSs has also been shown to exhibit DNA-helicase activity, but both activities do not appear to be required for its RSS functionality [24].

The NSs from groundnut ringspot virus (GRSV, NSs^GRSV^) and tomato yellow ring virus (TYRV, NSs^TYRV^), two distinct tospoviruses classified respectively in the American and Eurasian clades, have been shown to exhibit affinity for small and long dsRNA as well [20]. Furthermore NSs^GRSV^ also contains a WG/GW motif like NSs^TSWV^, but NSs^TYRV^ does not, while on the other hand both NSs^GRSV^ and NSs^TYRV^ contain a Walker motif A indicative of putative NTPase/phosphatase activity. Although most tospovirus NSs proteins have not yet been studied on their ability to suppress RNA silencing to the extent of NSs^TSWV^ and a generic mode of RNA silencing suppression is anticipated for all tospovirus NSs proteins, the presence or absence of motifs from certain NSs proteins raises the possibility of differences in their mode of action.

In the present study, a quantifiable system on systemic RNA silencing suppression was established, using transgenic *Nicotiana benthamiana* line 16c constitutively expressing GFP [9, 25], to comparitively analyse TSWV, GRSV and TYRV NSs proteins on suppression of systemic silencing. *N*. *benthamiana* 16c plants were chosen for the present study as the constitutive expression of GFP in their leaves/stem allow easier monitoring (under UV light) of systemic GFP silencing. This system was additionally employed to functionally analyze NSs proteins from silencing-compromised TSWV isolates (160 and 171) and NSs^TSWV^ gene constructs, mutated in predicted RNA binding domains, on their ability to suppress systemic GPF silencing.

## Results

### Establishment of a quantifiable system on suppression of systemic RNA silencing

Due to the affinity of tospoviral NSs for small si- and miRNAs [20], NSs is likely to prevent systemic spread of the RNA silencing signal. However, this has only been described to a very limited extent for NSs^TSWV^ [16] and extensive studies on this as well as other tospoviral NSs proteins are still lacking.

**Fig 1:**
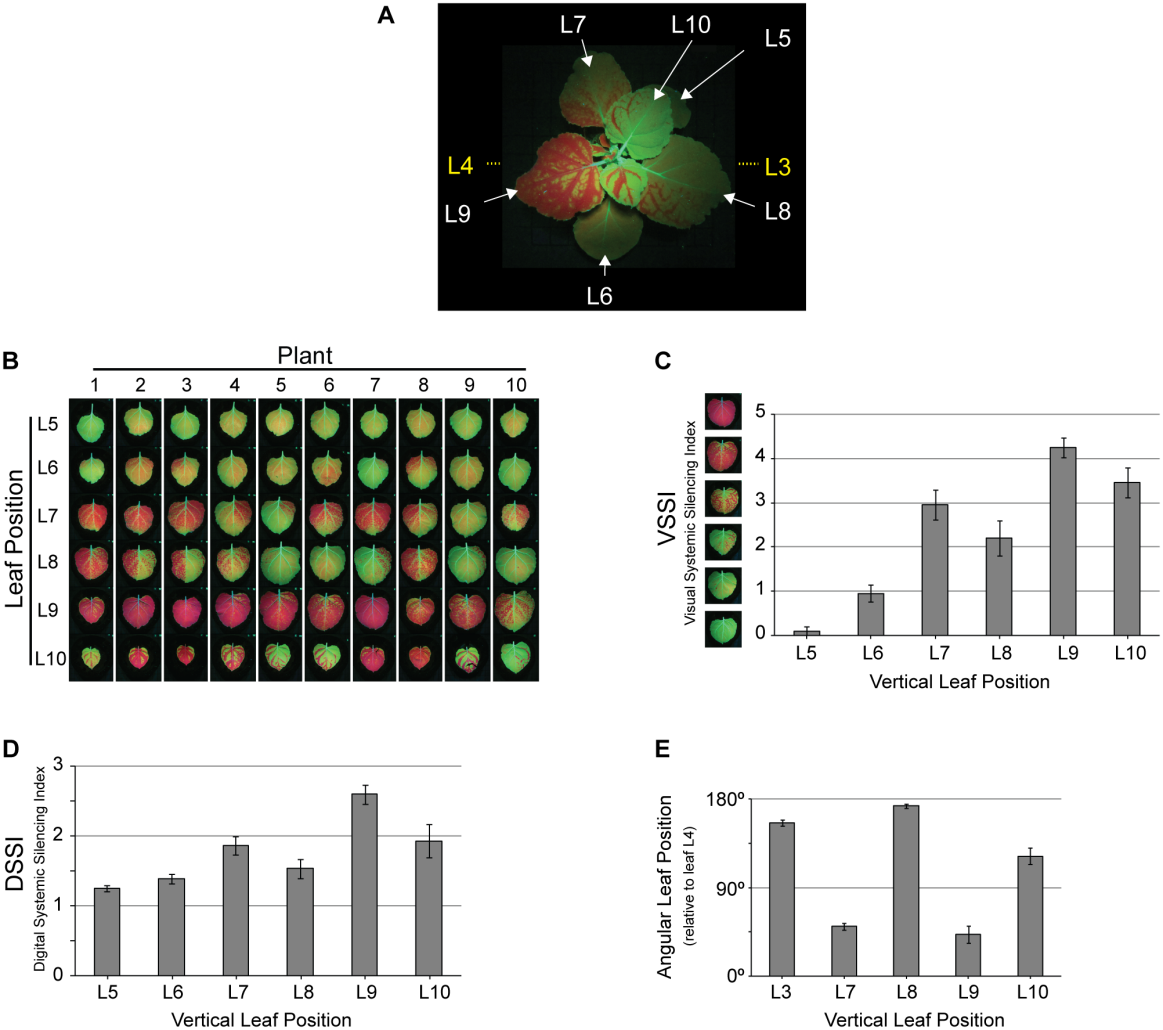
Systemic GFP silencing 17 days after agroinfiltration of leaves L3 and L4 with pBinGFP and pBinGUS. (A) Systemic GFP silencing in a *N*. *benthamiana* 16C plant, showing vertical leaf positions. White letters indicate the position of leaves analysed for systemic silencing. Agroinfiltrated leaves L3 and L4 are not visible, and their positions are indicated (yellow letters). (B) Visual overview on systemic GFP silencing in leaves L5—L10 from ten *N*. *benthamiana* 16C plants. (C) Visual Systemic Silencing Index (VSSI) of systemic GFP silencing in leaves shown in panel B. (D) Digital Systemic Silencing Index (DSSI) of systemic GFP silencing in leaves shown in panel B. (E) Angular leaf position (0–180 degrees) of leaves L7—L10 and the agroinfiltrated leaf L3 relative to the agroinfiltrated leaf L4 (set as reference on zero degrees). Error bars in panels C and D indicate the standard error of the mean (SEM) of measures resulting from 10 leaves.

To comparatively analyze various tospoviral NSs proteins, a quantifiable system on (suppression of) systemic silencing was developed. To this end, individual leaves of ten *N*. *benthamiana* 16C (GFP transgenic) were co-agroinfiltrated with pBinGFP and pBinGUS (as a non-RSS). At 17 days post agroinfiltration (dpa) (Fig 1A) the six leaves (L5, L6, L7, L8, L9, L10) above the infiltrated leaves (L3 and L4) of each plant were collected according to their respective vertical position. These leaves were visually scored regarding their level of systemic silencing, induced by the local infiltration of pBinGFP, using an arbitrary system here referred to as Visual Systemic Silencing Index (VSSI). Using this index, the leaves were categorized in six levels of systemic silencing that ranged from 0 (leaf with no systemic silencing) to 5 (leaf completely silenced) (S1 Fig). Based on the VSSI analysis, systemic GFP silencing was very weak in leaves L5 and L6 and consistently observed strongest in leaves at position L9 (Fig 1A, 1B and 1C). Although the VSSI approach worked nicely, like other systems that previously tried to quantify systemic silencing [14, 26], it entirely relied on a visual judgment and in case of only small differences mistakes can be easily made. To circumvent this problem, the Systemic Silencing Index (SSI) was digitalized (Digital Systemic Silencing Index—DSSI) by calculating the ratio of red (chlorophyll autofluorescence) / green (GFP fluorescence) measured by ImageJ analysis of digital pictures taken from leaves L5—L10 as described in Material and Methods. Using this approach, calculated DSSI values again showed highest systemic silencing scores for leaves at position L9, and furthermore were in agreement with the VSSI scores obtained from all the other leaves as well (Fig 1D).

Next, the amount of systemic GFP silencing observed in the upper leaves was investigated in relation to their angular distance relative to the (lower) agroinfiltrated leaves (Fig 1E and S2 Fig). To this end, the angular positions of the leaves that exhibited (strong) systemic silencing (L7—L10) were determined relative to leaf L4 (the youngest of the two infiltrated leaves) that was set at 0^°^. The second (oldest) infiltrated leaf (L3) was located about 180° from leaf L4. Since systemic GFP silencing was always absent or very weak in leaves L5 and L6, these leaves were left out during the remainder of the analysis. In light of its angular position, leaf L9 was closest to the infiltrated leaf L4, followed by leaf L7, while leaves L8 and L10 were respectively the first and second closest ones to the (oldest) infiltrated leaf L3 (Fig 1E and S2 Fig). According to these angular leaf positions, the systemic GFP silencing signal was always most strongly spread from leaf L4 (and not L3) and lead to strongest silencing in leaf L9. Based on these data, only leaf L4 was onwards infiltrated as standard for the induction of GFP silencing and leaf L9 analyzed for (suppression of) systemic GFP silencing.

### Dose-dependent suppression of systemic silencing by NSs^TSWV^

**Fig 2:**
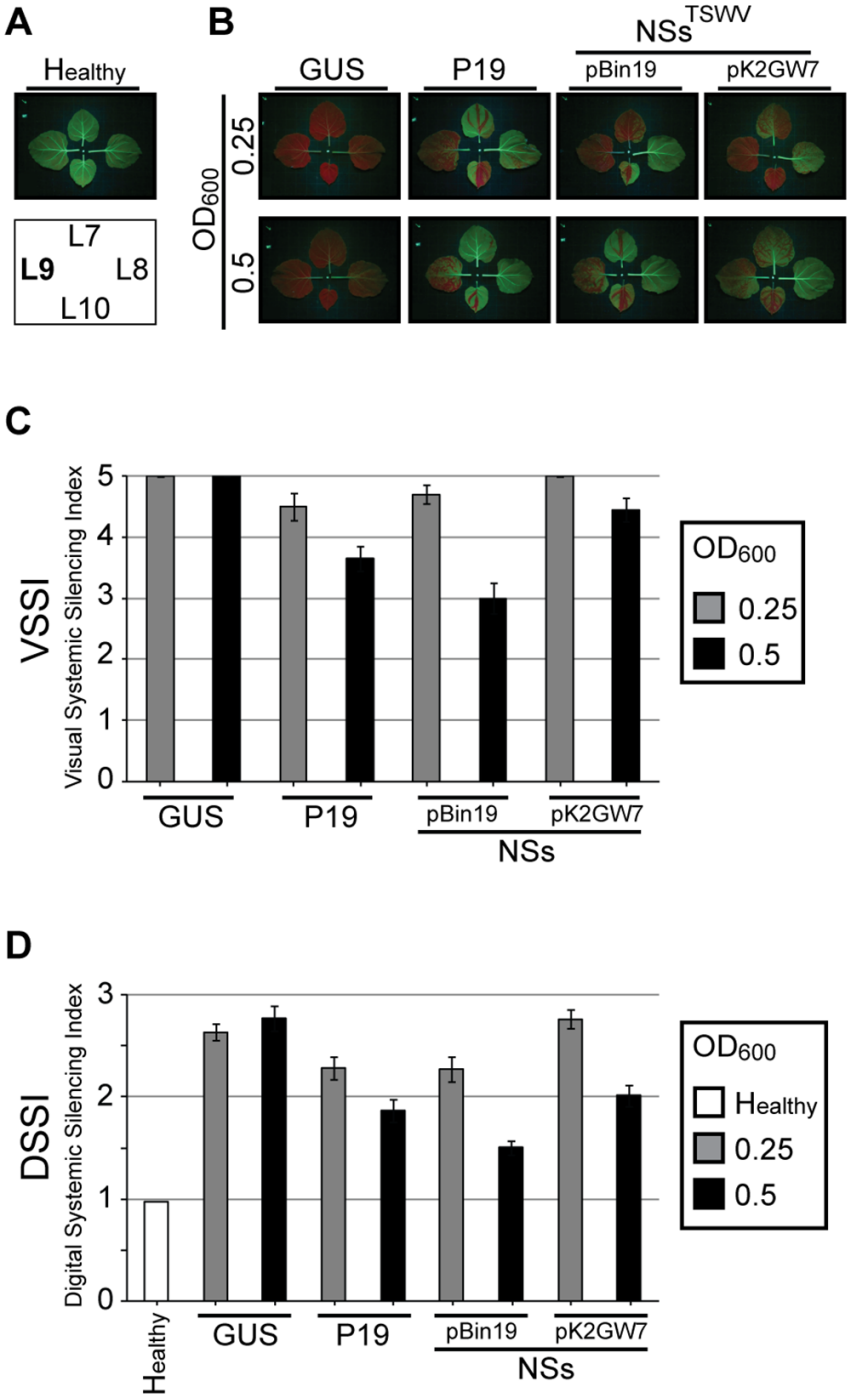
Dose-dependent suppression of systemic GFP silencing. *N*. *benthamiana* 16C plants were analysed at 17 days after co-agroinfiltration of leaf L4 with pBinGFP and various RSS gene constructs. (A) Upper panel show the four top leaves of non-infiltrated 16C *N*. *benthamiana*. Vertical leaf positions are indicated in the lower panel (arrangement does not reflect angular leaf positions). (B) Four top leaves of 16C plants co-agroinfiltrated (at leaf L4) with GFP and GUS or P19 or TSWV NSs (in vector pBin19 and pK2GW7) with different OD_600_ as indicated. (C) Visual Systemic Silencing Index (VSSI) of systemic GFP silencing in leaves L9 from plants as shown in panel B. (D) Digital Systemic Silencing Index (DSSI) of systemic GFP silencing in leaves L9 from plants as shown in panel B. Error bars indicate the standard error of the mean (SEM) of measures resulting from 10 leaves.

Earlier, a dose-dependent effect of the tombusvirus RSS P19 protein has been described [11], but nothing on this has been reported during studies that investigated suppression of systemic RNA silencing by transiently expressed viral RSS proteins. In several of these cases viral RSS were rather proposed to lack the ability to suppress systemic silencing or to do so very weakly [14, 27]. However, these results may also just reflect a dose-dependency in which the absent/weak systemic silencing suppression was simply caused by insufficient/low protein expression levels. To test this hypothesis, systemic GFP silencing was analyzed in the presence of varying amounts of transiently expressed NSs^TSWV^ using the established VSSI and DSSI systems as described above. To this end, L4 leaves from *N*. *benthamiana* 16C plants were co-agroinfiltrated with a fixed amount of *A*. *tumefaciens* suspension containing pBinGFP and varying amounts of *A*. *tumefaciens* (optical densities (OD) 0.25 and 0.5) containing binary constructs of NSs^TSWV^. Suppression of systemic GFP silencing was determined in leaves L9 by VSSI and DSSI. As positive and negative controls respectively P19 and GUS were included in the experiment. Since our experience during earlier experiments already indicated that transient NSs^TSWV^ expression levels were always higher from the (conventional) binary vector pBin19 compared to pK2GW7 (Gateway vector), experiments to demonstrate a dose-dependent suppression of systemic GFP silencing by NSs^TSWV^ were performed using both binary vectors. At 17 dpa GFP silencing was clearly visual in leaves L7 to L10 (Fig 2A and 2B), and the suppression of systemic GFP silencing was quantified by calculation of the VSSI and DSSI for leaf L9. Results showed that the level of systemic silencing in plants when GFP was co-expressed with GUS (negative control) was always in the same range regardless of the OD of the agrobacterium (GUS) suspension used. In contrast, when P19 or NSs^TSWV^ were co-infiltrated with pBinGFP, a clear dose-dependent suppression of systemic GFP silencing in L9 was observed, with increasing suppression levels when agrobacterium (P19 or NSs^TSWV^) suspensions with higher OD were infiltrated (Fig 2C and 2D and S4 Fig). Western immunoblot analysis in those cases confirmed the presence of higher amounts of NSs^TSWV^ (S3 Fig). As expected, the suppression of systemic silencing was also consistently stronger when NSs^TSWV^ was expressed from pBin19 compared to pK2GW7 (Fig 2C and 2D and S4 Fig) and correlated with higher protein expression levels from pBinNSs^TSWV^ (S3 Fig).

### GRSV and TYRV NSs, and their his-tagged versions, suppress local silencing

**Fig 3:**
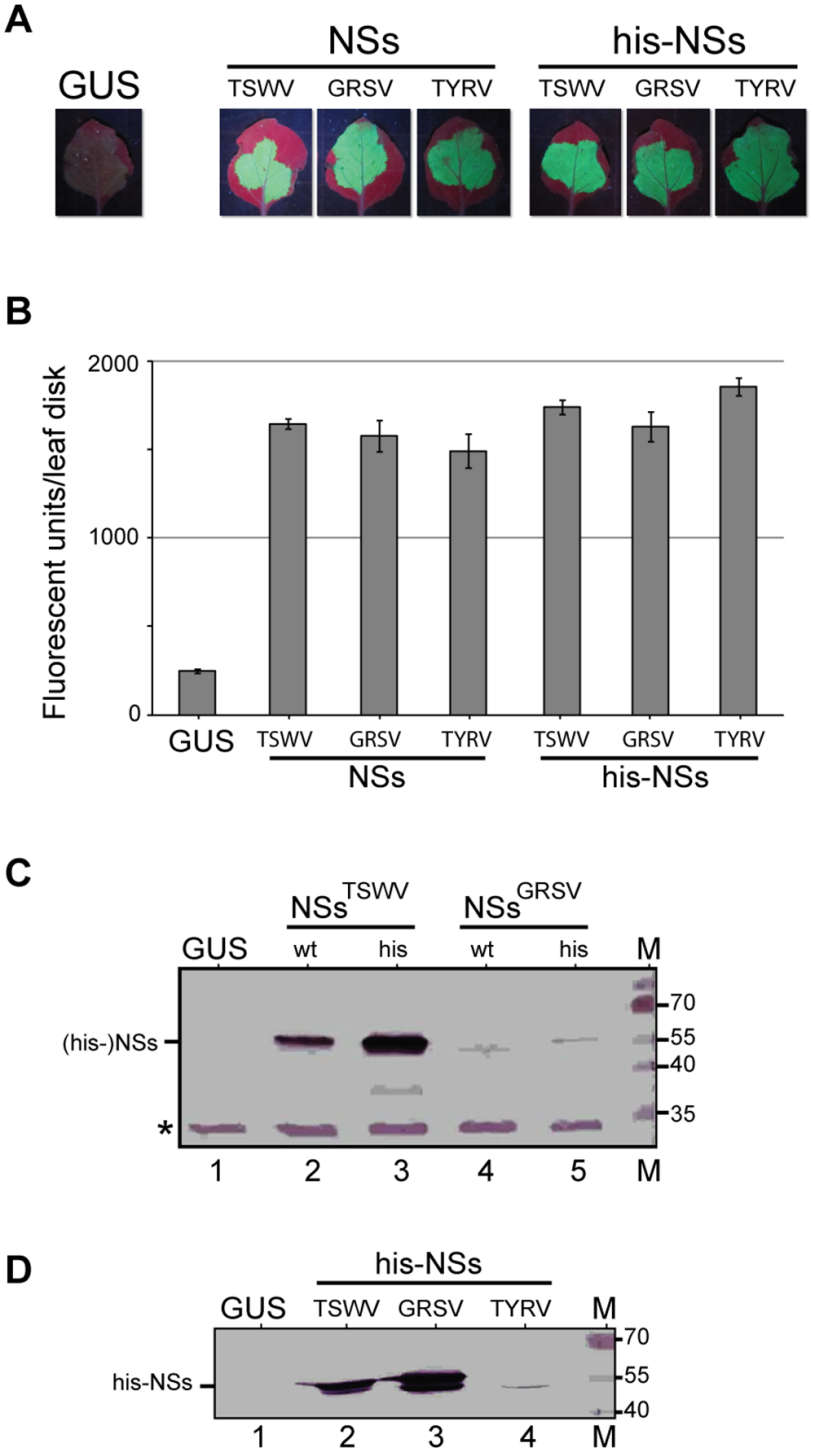
Suppression of local GFP silencing in *N*. *benthamiana* leaves. (A) Fluorescence images (5 dpi) from leaves co-infiltrated with pBinGFP and NSs gene constructs (TSWV, GRSV, TYRV) in binary vector pK2GW7. A leaf infiltrated with pBinGUS was included as control. (B) Number of fluorescence units [28] measured in leaf disks (1 cm^2^) collected from the agroinfiltrated leaf areas shown in panel A. Error bars indicate standard error of the mean (SEM) of three replicates. (C) Western immunoblot detection of TSWV and GRSV NSs (using antibody specific for TSWV NSs), and their corresponding his-tagged constructs, transiently expressed in *N*. *benthamiana*. A non-specific band (*) was used as loading control. (D) Western immunoblot detection of his-tagged constructs from TSWV, GRSV and TYRV NSs (same samples as panel C) using anti-polyhistidine antibody (Sigma Aldrich). Marker sizes are indicated at the right hand side.

Prior to a comparative analysis of TSWV, GRSV and TYRV NSs proteins on the suppression of systemic GFP silencing, NSs^GRSV^ and NSs^TYRV^ were first verified for their ability to suppress local GFP silencing. To this end, 35S-driven binary constructs of NSs^GRSV^ and NSs^TYRV^ were made and subsequently co-infiltrated into *N*. *benthamiana* with a construct containing a functional GFP. As positive and negative controls, constructs containing NSs^TSWV^ and GUS were included. As expected, the results showed that NSs^GRSV^ and NSs^TYRV^, like NSs^TSWV^, suppressed local GFP silencing (Fig 3A). The GFP fluorescence was also quantified as earlier described [28] and was slightly stronger in the presence of NSs^TSWV^ (Fig 3B). To verify that in all these leaves similar NSs expression levels were observed, extracts from the infiltrated leaf areas were analyzed by Western Immunoblotting. However, while NSs^TYRV^ was not efficiently detected by the monoclonal antibody against Asian tospovirus (data not shown), the additional use of different antisera for detection of NSs proteins, i.e. a polyclonal antiserum against TSWV NSs (anti-NSs^TSWV^) for detection of TSWV and GRSV NSs, and a monoclonal antibody against Asian tospovirus types of NSs for detection of TYRV NSs [29], did not allow a comparative analysis of the expression levels from different NSs proteins (Fig 3C, lanes 2, 4).

To quantify and comparatively analyze transient expression levels of NSs^TSWV^, NSs^GRSV^ and NSs^TYRV^, and correlate these to differences observed between local/systemic suppression of GFP silencing, N-terminal histidine(6)-fusion constructs were made and used. An earlier study already showed that a N-terminal his-tag fusion to the NSs^TSWV^ protein did not hamper its RNA silencing suppressor activity [20]. Constructs made were cloned into pK2GW7 and subsequently co-infiltrated with a functional GFP construct into *N*. *benthamiana* to analyze suppression of local GFP silencing. As positive and negative controls, respectively the untagged wild type NSs (TSWV, GRSV and TYRV) and GUS constructs were included. In the presence of GUS, local GFP expression was almost fully silenced at five dpa, while in the presence of his-NSs^TSWV^, his-NSs^GRSV^ and his-NSs^TYRV^ high levels of GFP expression were discerned (Fig 3A and 3B), indicating that all three proteins were able to suppress local GFP silencing to a similar extent and that the N-terminal his-tag did not abrogate RSS activity. Furthermore, RSS activity of all his-tagged NSs constructs was similar to the RSS activity of their untagged wild type constructs (Fig 3A). Upon fluorescence quantification, his-NSs^TSWV^ and his-NSs^TYRV^ consistently showed a slightly higher suppression of GFP silencing than its corresponding wild type constructs, while the levels of suppression of his-NSs^GRSV^ and NSs^GRSV^ were in the same range (Fig 3B). Western immunoblot analysis to verify for the levels of NSs expression using monoclonal anti-polyhistidine antibody (anti-his) (Sigma Aldrich) this time showed that the levels of his-NSs^TSWV^ and his-NSs^GRSV^ were similar while the one of his-NSs^TYRV^ was surprisingly much lower (Fig 3D). When the expression levels of the untagged and his-tagged NSs proteins of TSWV and GRSV were comparatively analysed using the polyclonal antiserum directed against NSs^TSWV^, the amount of his-NSs^TSWV^ was slightly higher compared to its untagged version (Fig 3C, lanes 2, 3), while the untagged and his-tagged NSs^GRSV^ were only weakly detected at similar levels (Fig 3C, lanes 4, 5). This weak detection was due to anti-NSs^TSWV^ antiserum cross-reacting only weakly with NSs^GRSV^, as supported by the observation that detection of his-NSs^GRSV^ using anti-his antibody showed expression levels of his-NSs^GRSV^ (Fig 3D, lane 3) similar to the expression levels of his-NSs^TSWV^ (Fig 3D, lane 2). Comparison between the expression levels of untagged and his-tagged NSs^TYRV^ was not possible since detection with monoclonal antibody against Asian tospovirus NSs proteins rendered unclear results (data not shown). However detection using anti-his antibody indicated relatively low levels of expression compared to those of TSWV and GRSV (Fig 3D).

### Comparative analysis of NSs^TSWV^, NSs^GRSV^ and NSs^TYRV^ on suppression of systemic silencing

**Fig 4:**
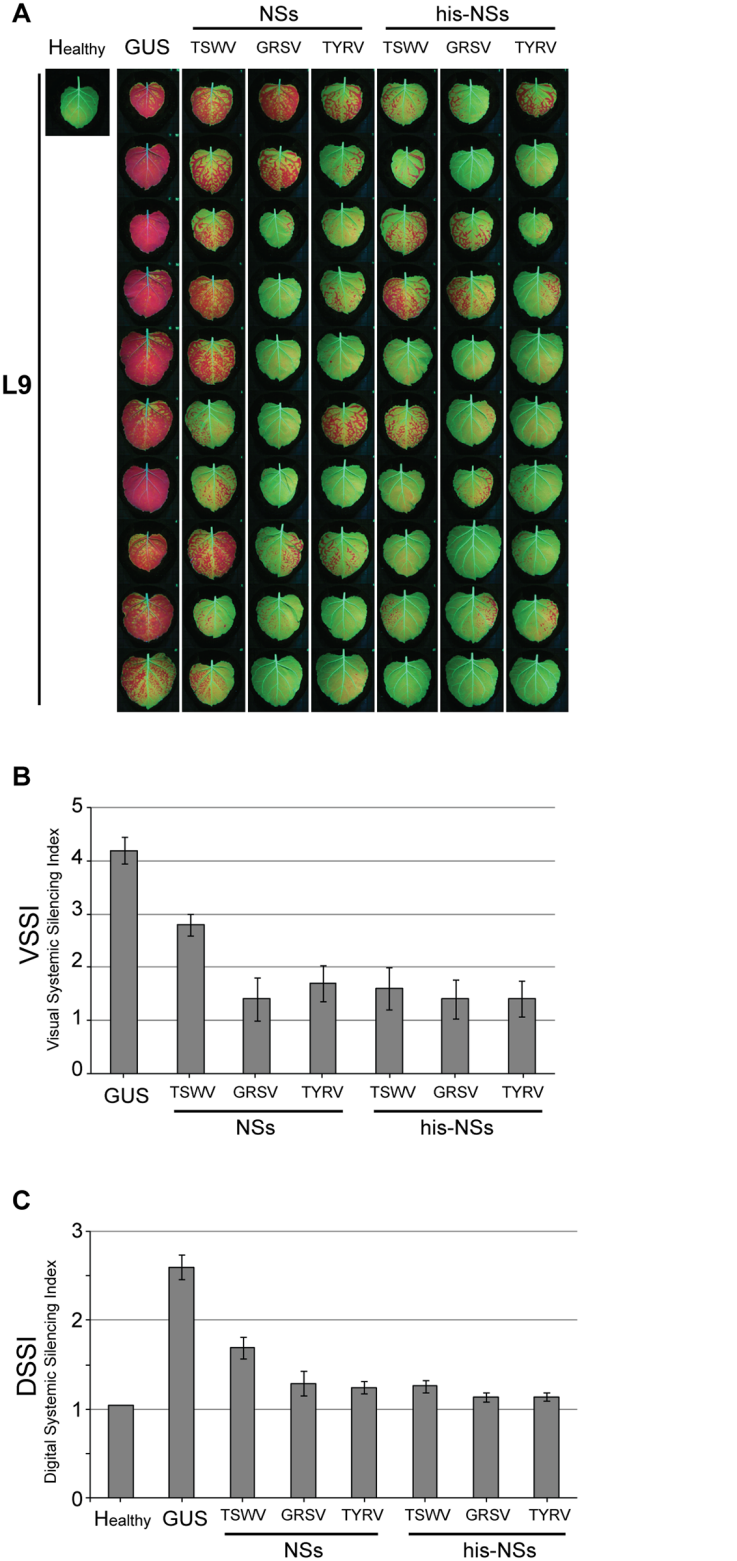
Systemic GFP silencing at 17 days after agroinfiltration with pBinGFP and various NSs gene constructs. *N*. *benthamiana* 16C plants were infiltrated at leaf L4 with pBinGFP in the additional presence of his-tagged NSs constructs from TSWV, GRSV or TYRV. As negative control, plants were infiltrated with pBinGUS. (A) Fluorescence images on systemic GFP silencing in leaves L9 from ten 16C plants in the presence of NSs gene constructs as indicated (B) Visual Systemic Silencing Index (VSSI) of systemic GFP silencing in leaves from panel A. (C) Digital Systemic Silencing Index (DSSI) of systemic GFP silencing in leaves from panel A. Error bars indicate the standard error of the mean (SEM) of measures resulting from 10 leaves.

Having demonstrated the local RSS activity of NSs^GRSV^ and NSs^TYRV^, their ability to suppress systemic silencing was analysed comparatively to NSs^TSWV^. When the untagged and his-tagged version of these NSs proteins were tested and quantified using the VSSI and DSSI systems described above, all were able to suppress systemic silencing (Fig 4A). However and interestingly, while all NSs proteins earlier showed similar levels of local GFP silencing suppression (Fig 3A and 3B), even though western immunoblot analysis showed lower expression levels for his-NSs^TYRV^ (Fig 3D), suppression of systemic GFP silencing by NSs^TSWV^ was slightly weaker compared to NSs^GRSV^ and NSs^TYRV^ (Fig 4A, 4B and 4C and S5 Fig). This weaker suppression of systemic silencing was not observed with his-NSs^TSWV^, which showed similar values as the other his-tagged NSs constructs tested (Fig 4B and 4C and S5 Fig). It is most likely that these differences were being caused by a (slight) difference in expression levels, since higher expression levels of NSs^TSWV^ were observed in the additional presence of the his-tag (Fig 3C, lanes 2, 3).

### Analysis of NSs^TSWV^ mutants on suppression of systemic silencing

**Fig 5:**
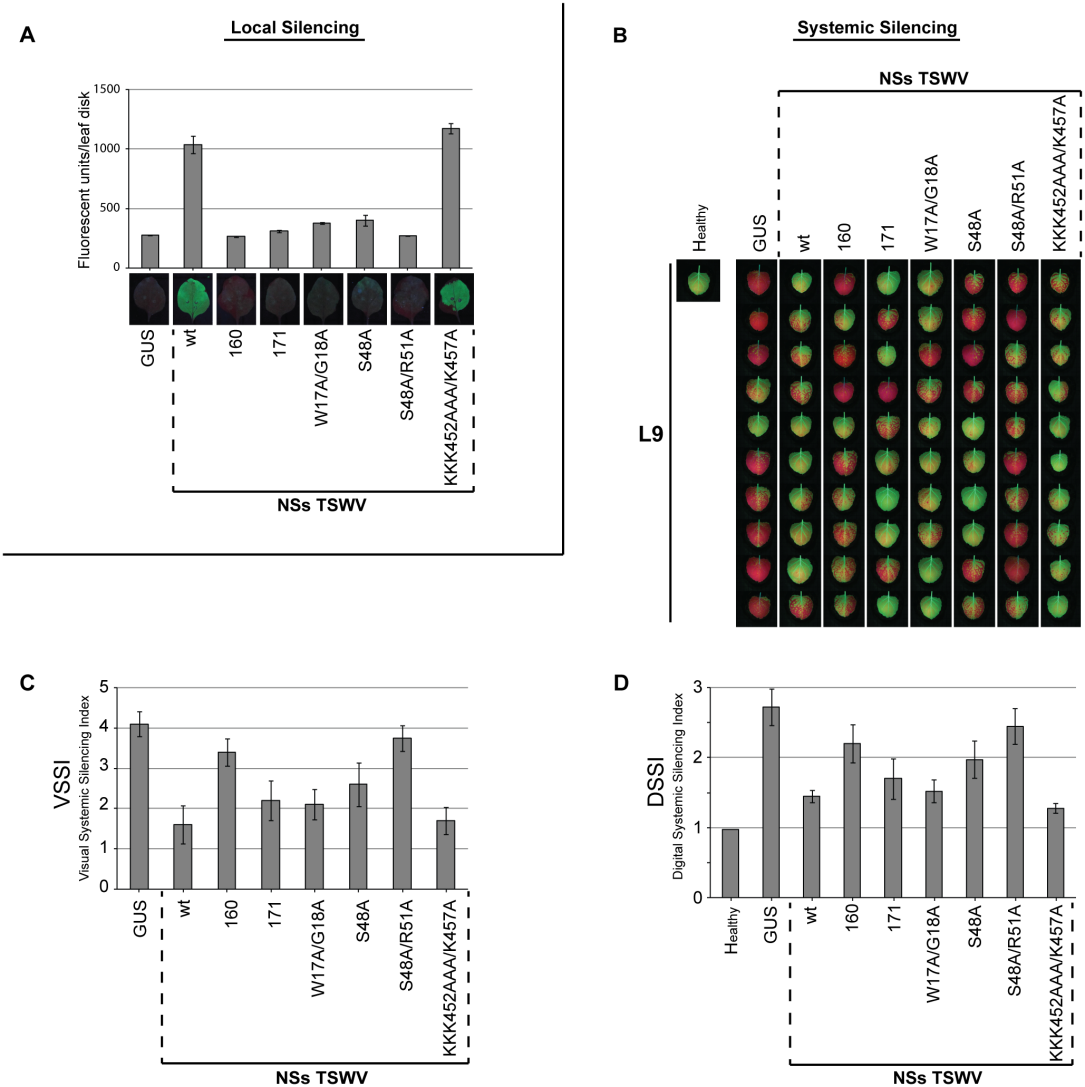
Systemic GFP silencing at 17 days after agroinfiltration with pBinGFP and various NSs mutants/variants. *N*. *benthamiana* 16C plants were infiltrated at leaf L4 with pBinGFP in the additional presence of NSs gene constructs from TSWV isolates 160 or 171, or from NSs^TSWV^ mutants W17A/G18A, S48A, S48A/R51A, KKK452AAA/K457A. As a negative control leaves were infiltrated with pBinGUS. As a positive control NSs from TSWV BR01 (indicated as “wt”) was used. (A) Fluorescence images (bottom) on local GFP silencing suppression in *N*. *benthamiana* leaves by the NSs mutant/variant gene constructs indicated. Graph shows the number of fluorescence units [28] measured in leaf disks (1 cm^2^) collected from the agroinfiltrated leaf areas. Error bars indicate standard error of mean (SEM) of three replicates. (B) Fluorescence images of systemic GFP silencing in leaves L9 from ten 16C plants in the presence of NSs muntat/variant gene constructs indicated. (C) Visual Systemic Silencing Index (VSSI) of systemic GFP silencing in leaves shown in panel B. (D) Digital Systemic Silencing Index (DSSI) of systemic GFP silencing in leaves shown in panel B. Error bars indicate the standard error of the mean (SEM) of measures resulting from 10 leaves.

Recently NSs^TSWV^ was identified as the avirulence (Avr) determinant of the single dominant *Tsw* resistance (R) gene, and an extensive alanine mutant screen of NSs revealed the importance of the amino-terminus in both RSS and Avr functionality [22, 28]. Using the established VSSI and DSSI systems, a selected set of four NSs^TSWV^ mutants, containing mutations in predicted RNA binding domains or a putative AGO1 interaction domain (Table 1), and two NSs variants from TSWV isolates (NSs^160^ and NSs^171^) able to break *Tsw* resistance and hampered in their local RSS activity [22, 28] were further analyzed for their ability to suppress systemic GFP silencing. Prior to this, all constructs were first verified for their ability to suppress local GFP silencing in *N*. *benthamiana*. In accordance to earlier data [22], only mutant NSs-KKK452AAA/K457A was able to suppress local silencing with similar strength to wt NSs^TSWV^. All other (mutant/variant) NSs constructs showed absent suppression of local GFP silencing (NSs^160^ and NSs-S48A/R51A) or only very low levels (NSs^171^, NSs-W17A/G18A, NSs-S48A) (Fig 5A).

**Table 1:** Comparison of RSS activity from NSs^TSWV^ mutants used in the present study.

		RSS activity ^c^	
Mutant ^a^	Mutation target ^b^	Local	Systemic
GUS	Negative control	**−**	**−**
NSs^TSWV^ (wt)	Positive control	**++**	**++**
NSs-W17A/G18A	Putative AGO1 interaction domain	**+/−**	**++**
NSs-S48A	Predicted RNA-binding domain	**+/−**	**+**
NSs-S48A/R51A	Predicted RNA-binding domain	**−**	**+/−**
NSs-KKK452AAA/K457A	Predicted RNA-binding domain	**++**	**++**

GUS, beta-glucuronidase; wt, wild type; AGO1, Argonaute 1; RSS, RNA silencing suppressor.

^a^ Mutants are ordered according to mutated amino acid residue numbered from the amino-terminal end.

^b^ Predicted function of the mutated amino acid [22].

^c^ RNA silencing suppression strength (relative to NSs^TSWV^ wild type): absent (−), weak (+/−), mild (+), strong (++).

When all NSs constructs were next tested on their ability to suppress systemic GFP silencing using the VSSI and DSSI systems, all four alanine substitution NSs mutants were still able to suppress systemic silencing. However, a more detailed look showed that NSs mutants S48A and S48A/R51A exhibited only low levels of systemic silencing suppression while NSs mutants W17A/G18A and KKK452AAA/K457A were about as strong as the wild type NSs^TSWV^ (Fig 5B, 5C and 5D and S6 Fig). Furthermore, mutant NSs-S48A/R51A was even more compromised in the ability to suppress systemic GFP silencing compared to its single mutant NSs-S48A (Fig 5B, 5C and 5D and S6 Fig). From the two resistant breaker isolates, NSs^171^ was able to suppress systemic silencing, less than wt NSs^TSWV^, but more than NSs^160^, which was more compromised (Fig 5B, 5C and 5D and S6 Fig).

**Fig 6:**
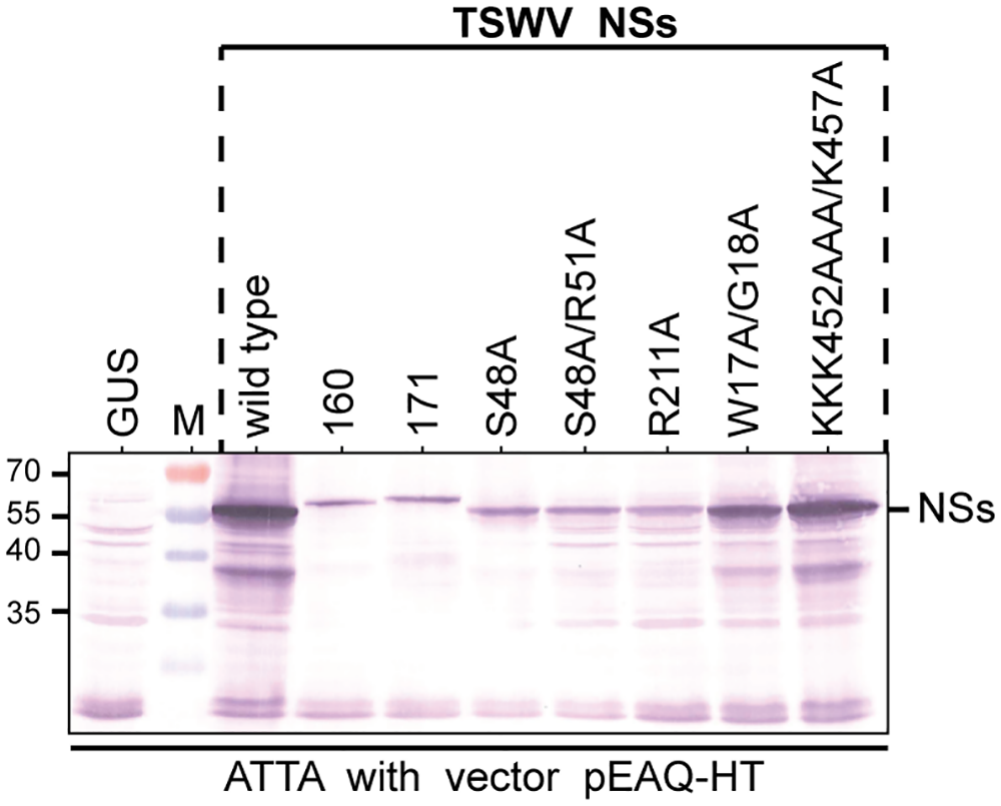
Western immunoblot detection of TSWV NSs from NSs wild type/mutant/variant gene constructs. Translatability of NSs gene constructs from isolates BR01 (wild type), resistance breaker isolates 160 and 171 and mutants W17A/G18A, S48A, S48A/R51A, KKK452AAA/K457A was verified in leaf samples from *N*. *benthamiana* agroinfiltrated with agrobacterium harboring these NSs constructs in binary vector pEAQ-HT (which co-expresses tombusviral RSS P19). Similar amounts (weight/volume) were loaded on SDS-PAGE and detected by western immunoblotting using a polyclonal antiserum specific to TSWV NSs. Size markers are indicated at the left hand side.

To rule out that the absence of suppression of silencing was due to non-translatability of the (mutant) NSs constructs, their expression was verified by western immunoblotting. Due to low expression levels of some NSs mutants, likely due to a loss of RSS activity, their detection was difficult and to solve this problem the NSs constructs therefore were co-expressed with P19 RSS. All NSs constructs were expressed but only weakly, with the exception of NSs-W17A/G18A and NSs-KKK452AAA/K457A that showed somewhat similar expression levels compared to wt NSs^TSWV^ (Fig 6). The expression levels of NSs^171^ and NSs^160^ were earlier tested and correlated to their local suppression strength [28].

## Discussion

So far, studies on the mode by which tospovirus NSs is able to suppress RNA silencing have mostly been limited to its representative, *in casu*, TSWV. Here, it is shown that NSs^GRSV^ and NSs^TYRV^, like NSs^TSWV^, are able to suppress local and systemic silencing, supporting the idea that this is a generic feature for the NSs protein from members of the *Tospovirus* genus. Furthermore, evidence is presented indicating that the NSs^TSWV^ protein is able to suppress RNA silencing at another step further downstream siRNA sequestration. This is best demonstrated by the results obtained with NSs mutant W17A/G18A and NSs from the resistance breaking TSWV 171 isolate, both of which are clearly hampered in their local RNA silencing suppressor activity while they are still able to suppress systemic RNA silencing.

Short interfering RNA molecules play a major role in local and systemic silencing as in both cases they are needed to activate an antiviral RISC. Systemic silencing however requires that siRNAs prior to this have systemically moved as a mobile signal in order to activate RISC in systemic tissues [30]. Viral RSS proteins that are able to sequester siRNAs, like the tospovirus NSs protein [20], thus will not only prevent their uploading into RISC but also their systemic movement. A recent mutant screen of TSWV NSs that aimed to identify potential RNA binding domains [22] revealed the importance of the N-terminal part of NSs for RSS activity and avirulence (triggering of the dominant *Tsw* resistance gene). From this screen three NSs mutants (S48A, S48A/R51A and KKK452AAA/K457A) that mapped within two predicted RNA binding domains were further analyzed here using the established VSSI/DSSI systems. It was anticipated that in case essential RNA binding domains would be hit, those mutants would score negative on the ability to suppress systemic silencing. While mutants S48A and S48A/R51A partly or failed to suppress local RNA silencing, they showed respectively a mild and a weak suppression of systemic RNA silencing. In contrast, mutant KKK452AAA/K457A was still able to strongly suppress local and systemic RNA silencing. These data supported the idea that the first two mutants were likely affected by a genuine loss of RNA binding, while the third mutant was not, and suggested that its mutated sequence (KKKK452/K457) does not play a major role in RNA binding domain. A closer look at mutants S48A and S48A/R51A also indicated that the combined mutations of S48 and R51 were more detrimental to the ability to suppress local and systemic silencing than mutation of only S48. To further confirm the ability—or loss—of RNA binding by any of these NSs mutants, electrophoretic mobility shift assays were performed earlier but these failed due to their relatively low expression levels [22].

The fourth NSs mutant analyzed was changed at residues W17/G18, a motif that is not part of a predicted RNA binding domain [22]. GW/WG-motifs are known to function as an argonaute (AGO) hook in some RSS proteins, and promote their interaction with AGO proteins to inhibit RISC activity [31], as earlier demonstrated for turnip crinkle virus (TCV) P38 [32] and sweet potato mild mottle virus (SPMMV) P1 [21]. While the NSs-W17A/G18A mutant had lost most of its ability to suppress local RNA silencing it was still able to suppress systemic silencing at a level comparable to the wt NSs protein. This firstly indicated that NSs W17A/G18A was still preventing siRNAs to move systemically and activate the non-cell autonomous RNA silencing pathway. Secondly, and more interesting, its ability to suppress systemic silencing likely by binding siRNAs, while not being able to suppress local RNA silencing, indicated that residues W17/G18 are involved in another mode of action downstream of siRNA biogenesis and sequestration. Although these findings provide further support for a putative interaction with AGO1, further experiments are needed to provide proof for the existance of genuine NSs-AGO interactions.

Besides the four NSs mutants, two additional NSs variants collected from *Tsw* resistance breaking TSWV isolates (NSs^171^ and NSs^160^) were analyzed on their ability to suppress systemic silencing. Earlier, it was shown that these proteins exhibited no (NSs^160^) or only a weak (NSs^171^) ability to suppress local RNA silencing [28], but here it is shown that both are still able to suppress systemic silencing. While NSs^160^ showed low levels of systemic silencing suppression, NSs^171^ exhibited similar levels as the ones from NSs-W17A/G18A. These observations are in agreement with data from the NSs mutant screen [22] that have demonstrated that RSS activity and avirulence are two features of NSs that are not functionally coupled. Any mutation in the NSs protein that leads to a loss of avirulence, allowing the corresponding viruses to break *Tsw* resistance, thus not necessarily affect its additional ability to suppress (local and/or systemic) RNA silencing. In some cases it does affect only partially and/or locally, while leaving its ability to suppress systemic silencing unaltered, like in the case of the *Tsw* resistance breaker isolate NSs^171^ and mutant NSs-W17A/G18A. A further look at the amino acid sequences of NSs^171^ and NSs^160^ revealed a single nucleotide polymorphism (proline) in NSs^160^ at position S48, which is in a predicted RNA binding domain [22], that may have caused for its reduction in the level to suppress systemic silencing to a larger extent than the NSs^171^ (which does not harbor this S48P modification).

The analyses of NSs mutants/variants on their ability to suppress systemic silencing has made use of two newly developed systemic silencing index (SSI) assays, one that relies on visual index (VSSI) and a second one on a digital index (DSSI). Both indexes provide a way to study and compare the suppression of systemic silencing by different viral RSS proteins in a fixed experimental setting that only requires the infiltration of one leaf (L4) and a score based on a single systemic leaf (L9). Whereas the visual index (VSSI) allows a faster categorization and can be performed when having no access to ImageJ-like analysis tools, like all other quantification systems previously described [14, 26], it relies completely on a judgment by the observer. The digital index (DSSI) developed here, on the other hand, is unbiased and more accurate. In support of both indexes, however, results obtained during our entire investigation with each of them were always in close agreement with each other. Regardless of the chosen system, suppression of systemic silencing by NSs^TSWV^ was shown to be dose-dependent and in agreement with earlier indications on this [20]. A dose-dependent suppression of RNA silencing has earlier been shown in local assays for a few other viral RSS proteins that act by siRNA sequestration, including tombusvirus P19 and closterovirus P21 [4]. Here, this has now also been demonstrated for NSs^TSWV^ suppression of systemic silencing and stresses the importance of being more cautious when viral RSS proteins fail to suppress systemic silencing [14, 27] as those results might simply be due to low/insufficient RSS expression levels. A dose-dependent suppression of systemic silencing also makes sense in light of the idea that viral RSS proteins contribute to the severity of plant viral infections [33]. For TSWV this supports earlier observations on infections with a range of different TSWV isolates in which higher levels of NSs expression were often observed to correlate with more severe disease symptoms [34].

During the comparative analysis of tospoviral NSs proteins it was interesting to see that NSs^TSWV^ was somewhat more strongly expressed when fused with a his-tag at its N-terminus, and as a result led to a higher level of RNA silencing suppression. Another intriguing result was the observation that his-NSs^TYRV^ was only expressed at relatively low levels compared to his-NSs^TSWV^ and his-NSs^GRSV^ but still exhibited a strong ability to suppress local and systemic silencing. The reason for this is unclear. Although TYRV belongs to the Eurasian clade of tospoviruses and is more distantly related from American clade tospoviruses (TSWV and GRSV) [18, 35], a different mode of action is not expected considering their similar pathogenicity on *N*. *benthamiana*. However, when compared to results from his-NSs^TSWV^ and his-NSs^GRSV^, the observation that his-NSs^TYRV^ has lower expression but similar RNA silencing suppression strength implies it has a more efficient strategy or an alternative mode of action to suppress silencing, e.g. a stronger affinity to (short and long) dsRNA or the ability to target a step that is not efficiently targeted by TSWV and GRSV NSs. This, however, still remains to be further investigated.

In conclusion, here we have established a new and quantifiable systemic silencing system to investigate the suppression of systemic RNA silencing and demonstrated a dose-dependent suppression by viral RSS proteins. Combined with data from local silencing suppression assays this system will be very useful for initial characterization of RSS proteins and providing further support for the identification of predicted RNA binding domains. Based on data from a selected set of NSs mutants and variants we have also obtained further evidence that point towards the ability of TSWV NSs to interfere in the RNA silencing pathway further downstream siRNA biogenesis and sequestration and in which residues W17/G18 may play an important role.

## Materials and Methods

### Plants and agrobacterium strains

*Nicotiana benthamiana* and a GFP transgenic 16C line of *N*. *benthamiana* [9, 25] were grown at 24°C under 16 h / 8 h day/night regime. For agroinfiltration assays, *Agrobacterium tumefaciens* strain COR308 [36, 37] was used.

### Agrobacterium mediated transient expression assay (ATTA)

Transient expression assays were performed by agroinfiltration of binary vector gene constructs in *N*. *benthamiana*. To this end, *A*. *tumefaciens* were transformed with the binary expression vectors and a single colony grown overnight (28°C, 180 rpm) in LB3 medium (10 g/L tryptone, 5 g/L yeast extract, 4 g/L NaCl, 1 g/L KCl, 3 g/L MgSO_4_.7H_2_O) under proper antibiotics selection pressure (100 μg/ml kanamycin (pBin19) or 250 μg/ml spectinomycin (pK2GW7), and 2 μg/ml tetracycline). From the overnight culture, 600 μL were inoculated in 3ml of induction medium (10.5 g/L K_2_HPO_4_, 4.5 g/L KH_2_PO_4_, 1 g/L (NH_4_)_2_SO_4_, 0.5 g/L Sodium Citrate Dihydrate, 0.25 g/L MgSO_4_, 0.2% (w/v) glucose, 0.5% (v/v) glycerol, 50 mM acetosyringone and 10 mM MES pH5.6) and incubated overnight at 28°C, while shaking at 180 rpm. The next day, cells were pelleted at 4000 rpm for 15 min. and resuspended in Murashige-Skoog (MS) medium to an optical density at 600 nanometer (OD_600_) of 1.0 or 0.5. Agroinfiltrations were performed at the basal (abaxial) side of leaves.

### Constructs for transient expression

Binary vector pBin19 constructs with GFP, tombusviral P19 and tospoviral NSs^TSWV^ [15, 38], as well as pK2GW7 constructs with NSs^TSWV^, NSs^GRSV^, NSs^TYRV^ [20] were described earlier. Constructs for 6xhisNSs^TSWV^, 6xhisNSs^GRSV^, 6xhisNSs^TYRV^, GUS were generated by polymerase chain reaction (PCR) amplification using specific primers to introduce the 6xhis-tag sequence at the 5’end of the gene. The his-NSs coding sequences were cloned in GATEWAY vector pK2GW7 [39] using GATEWAY technology (Life Technologies). Binary vectors pK2GW7 and pEAQ-HT constructs with NSs^TSWV^ gene from isolates BR01 (wild type), resistance breaker isolates 160 and 171 and mutants W17A/G18A, S48A, S48A/R51A, KKK452AAA/K457A were previously described [22, 28]

### GFP systemic silencing assays in *N*. *benthamiana* 16C

For the induction of systemic GFP silencing, 3–4 weeks old seedlings of *N*. *benthamiana* 16C constitutively expressing GFP [9] were agroinfiltrated with pBinGFP. Leaves were numbered with the first leaf above the cotyledon being denoted L1, while the second leaf was denoted L2 and so on (Fig 1A). Agroinfiltration was performed in leaves L3 and L4 and plants were monitored during 20 days for the presence of systemic silencing. For each experiment, at least one repetition was performed.

### Systems for quantifiable analysis of systemic GFP silencing

In each experiment, 10 plants *N*. *benthamiana* 16C were agroinfiltrated with pBinGFP for the induction of systemic GFP silencing in the absence or additional presence of a binary vector NSs (mutant/variant) gene construct (previous section). Determination of the Visual Systemic Silencing Index (VSSI) was as follows: systemic silencing in leaves was visually classified into six levels (S1 Fig) and ranged from systemic silencing being absent (level 0) to vein restricted and localized in a few veins (level 1), vein restricted and spread into a group of connecting veins (level 2), mostly vein restricted with initial spread to leaf lamina (level 3), almost complete (level 4) and complete (level 5). The average of the systemic silencing index used was calculated from 10 plants as well as the standard error of the mean (SEM). In all systemic silencing experiments, leaves from one plant *N*. *benthamiana* 16C not agroinfiltrated (healthy) were also analysed with the Systemic Silencing Indexes, as a background control.

Determination of the Digital Systemic Silencing Index (DSSI) was performed by digital analysis of pictures taken from the four most top leaves (in analogy, denoted L7, L8, L9, L10) (Fig 1A). Digital pictures were taken from leaves using a Canon PowerShot A3200 IS and subsequently analysed using ImageJ. Levels of GFP silencing were analysed by calculation of the red and green ImageJ channels from the entire L7, L8, L9 and L10 leaves using the Digital Systemic Silencing Index (DSSI) script (file SCRIPT DSSI; available upon request). The values from the red channel (detecting the red fluorescence from chlorophyll) were divided by the values of the green channel (detecting the green GFP fluorescence), resulting in a DSSI value reflecting the level of systemic silencing. High DSSI values indicate strong systemic silencing, while low DSSI values indicate weak systemic silencing.

### Western immunoblot detection of NSs

Western immunoblot analysis was performed as previously described [28]. Detection of untagged and his-tagged NSs was done by a polyclonal antiserum specific to TSWV NSs [40], a monoclonal antibody specific to WSMoV NSs [29, 41] or a monoclonal antibody specific to polyhistidine (Sigma Aldrich). Preparation of samples for western immunoblot analysis was performed using one gram of agroinfiltrated leaf material as earlier described [28].

## Supporting Information

S1 Fig Definition of levels for the Visual Systemic Silencing Index (VSSI) to visually quantify systemic GFP silencing in individual leaves. (TIF)

S2 Fig Angular leaf positioning in *N*. *benthamiana*. Angular leaf position (0–360 degrees) of leaves L7—L10 and the agroinfiltrated leaf L3 relative to agroinfiltrated leaf L4 (which was the reference and set at zero degrees).
(TIF)

S3 Fig Western immunoblot detection of TSWV NSs transiently expressed (in *N*. *benthamiana* leaves) from pBin19 and pK2GW7, each using OD_600_ of 0.25 and 0.5. Detection was performed using antiserum against TSWV NSs. Marker sizes are indicated at the left hand side.
(TIF)

S4 Fig VSSI data from two additional repetitions on dose-dependent suppression of systemic GFP silencing. *N*. *benthamiana* 16C plants were agroinfiltrated at leaf L4 with GFP and GUS or P19 or TSWV NSs (in vector pBin19 and pK2GW7) with different OD_600_ as indicated. Leaf L9 was visually scored 17 days after agroinfiltration. Error bars indicate the standard error of the mean (SEM) (n = 15).
(TIF)

S5 Fig VSSI data from two additional repetitions on the suppression of systemic GFP silencing in *N*. *benthamiana* 16C at 17 days after agroinfiltration with pBinGFP and NSs^TSWV^, NSs^GRSV^ or NSs^TYRV^. Agroinfiltration was performed on leaf L4. Leaf L9 was visually scored 17 days after agroinfiltration. Error bars indicate the standard error of the mean (SEM) (n = 15).
(TIF)

S6 Fig VSSI and DSSI data from a repetition on systemic GFP silencing in *N*. *benthamiana* 16C at 17 days after agroinfiltration with pBinGFP and various NSs mutants/isolates. Agroinfiltration was performed on leaf L4. Leaf L9 was visually and digitally scored 17 days after agroinfiltration. Error bars indicate the standard error of the mean (SEM). For each NSs construct, 15 plants were agroinfiltrated, and seven plants were agroinfiltrated with GUS.
(TIF)
