# Supplementary figures and images for: Analysis of Tospovirus NSs Proteins in Suppression of Systemic Silencing
Source: PLoS One. 2015 Aug 14;10(8):e0134517. doi: 10.1371/journal.pone.0134517 (PMC4537313; doi:10.1371/journal.pone.0134517)

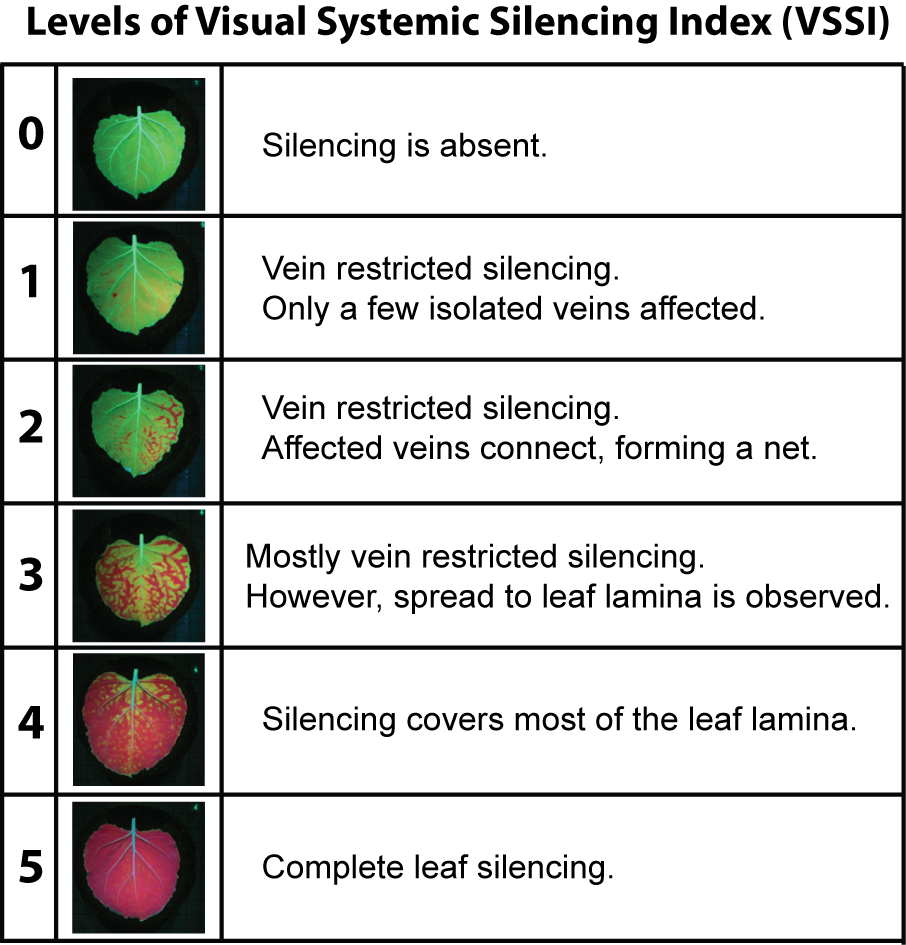

Supplement: S1 Fig — (TIF) [file pone.0134517.s001.tif]

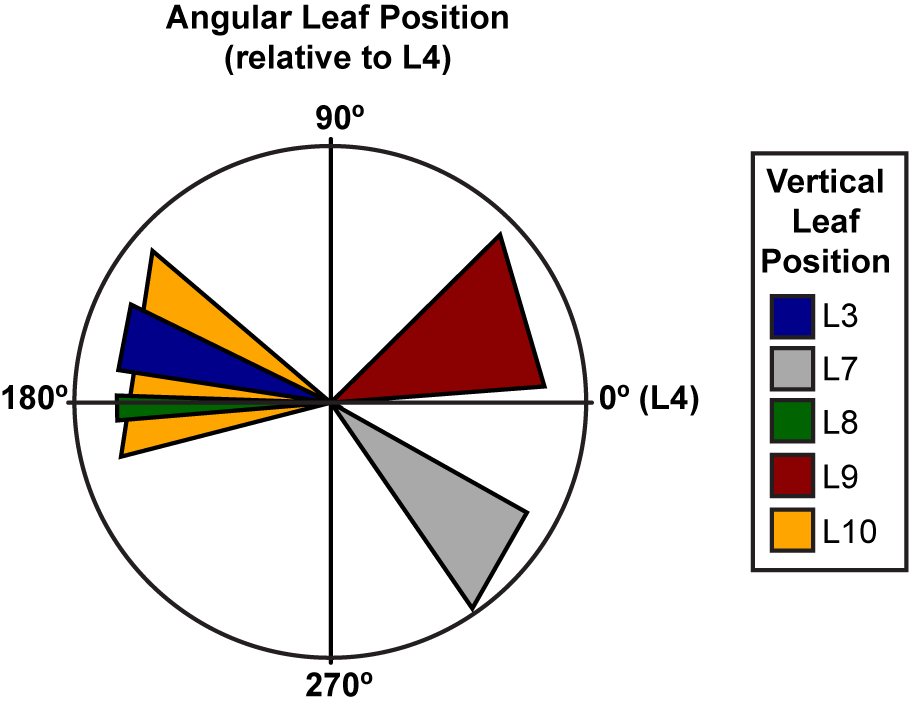

Supplement: S2 Fig — Angular leaf position (0–360 degrees) of leaves L7—L10 and the agroinfiltrated leaf L3 relative to agroinfiltrated leaf L4 (which was the reference and set at zero degrees). (TIF) [file pone.0134517.s002.tif]

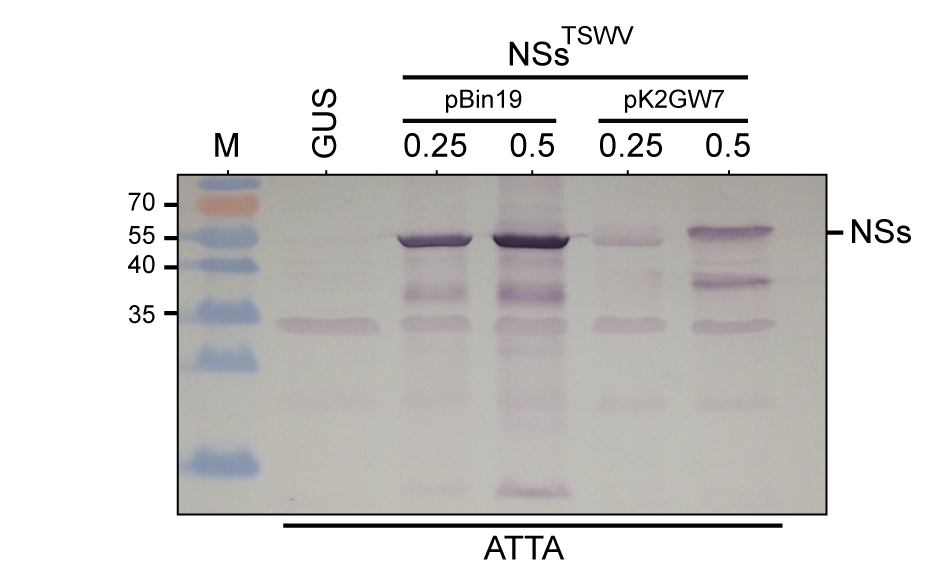

Supplement: S3 Fig — Detection was performed using antiserum against TSWV NSs. Marker sizes are indicated at the left hand side. (TIF) [file pone.0134517.s003.tif]

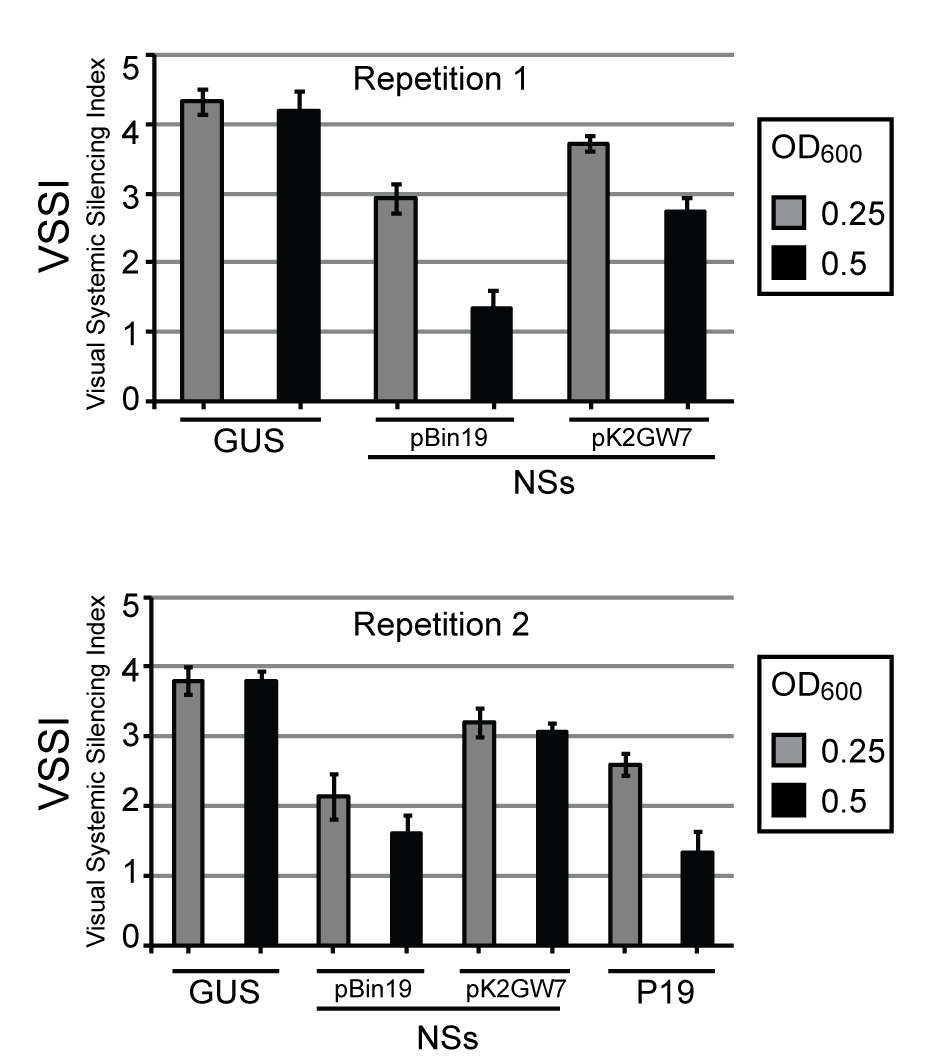

Supplement: S4 Fig — N. benthamiana 16C plants were agroinfiltrated at leaf L4 with GFP and GUS or P19 or TSWV NSs (in vector pBin19 and pK2GW7) with different OD600 as indicated. Leaf L9 was visually scored 17 days after agroinfiltration. Error bars indicate the standard error of the mean (SEM) (n = 15). (TIF) [file pone.0134517.s004.tif]

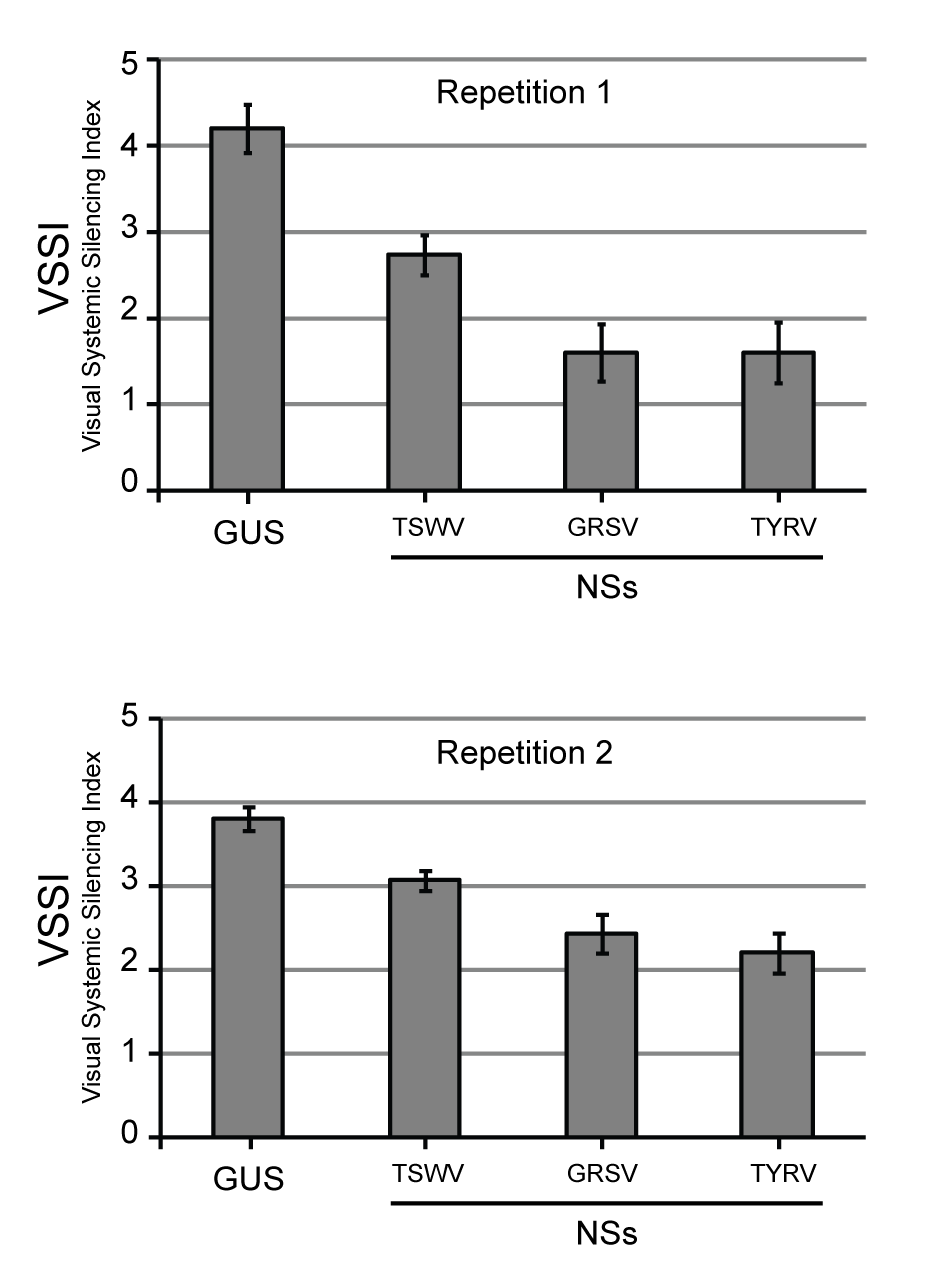

Supplement: S5 Fig — Agroinfiltration was performed on leaf L4. Leaf L9 was visually scored 17 days after agroinfiltration. Error bars indicate the standard error of the mean (SEM) (n = 15). (TIF) [file pone.0134517.s005.tif]

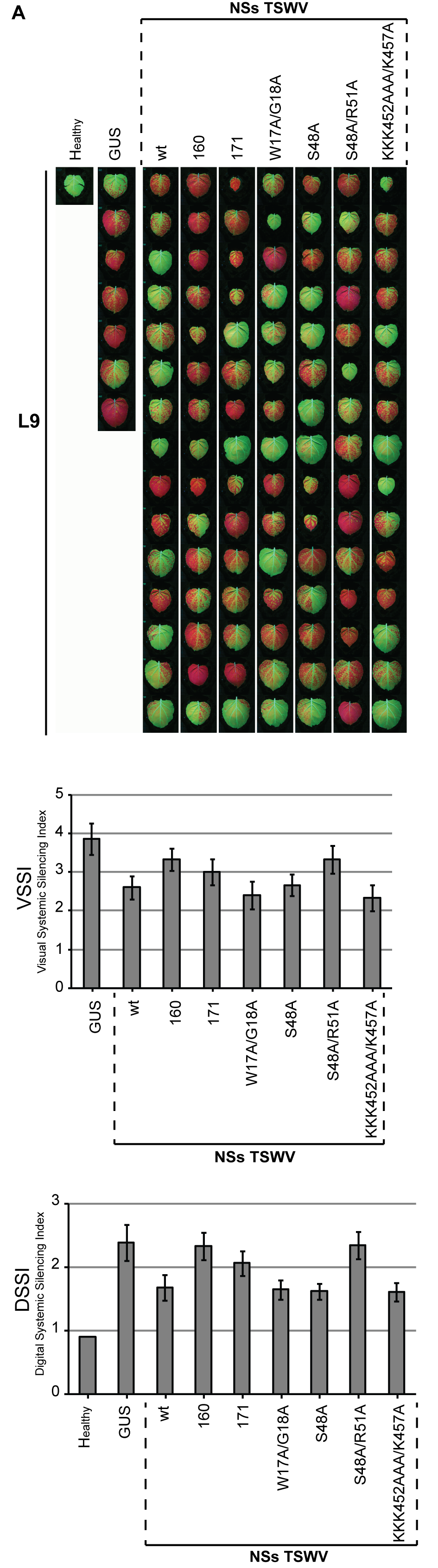

Supplement: S6 Fig — Agroinfiltration was performed on leaf L4. Leaf L9 was visually and digitally scored 17 days after agroinfiltration. Error bars indicate the standard error of the mean (SEM). For each NSs construct, 15 plants were agroinfiltrated, and seven plants were agroinfiltrated with GUS. (TIF) [file pone.0134517.s006.tif]
